# A biologically inspired decision-making system for the autonomous adaptive behavior of social robots

**DOI:** 10.1007/s40747-023-01077-5

**Published:** 2023-05-29

**Authors:** Marcos Maroto-Gómez, Álvaro Castro-González, María Malfaz, Miguel Ángel Salichs

**Affiliations:** Systems Engineering and Automation, University Carlos III of Madrid, Butarque 15, 28911 Leganés, Madrid Spain

**Keywords:** Autonomous decision-making, Social robots, Action selection, Adaptive behavior, Human–Robot Interaction

## Abstract

The decisions made by social robots while they fulfill their tasks have a strong influence on their performance. In these contexts, autonomous social robots must exhibit adaptive and social-based behavior to make appropriate decisions and operate correctly in complex and dynamic scenarios. This paper presents a Decision-Making System for social robots working on long-term interactions like cognitive stimulation or entertainment. The Decision-making System employs the robot’s sensors, user information, and a biologically inspired module to replicate how human behavior emerges in the robot. Besides, the system personalizes the interaction to maintain the users’ engagement while adapting to their features and preferences, overcoming possible interaction limitations. The system evaluation was in terms of usability, performance metrics, and user perceptions. We used the Mini social robot as the device where we integrated the architecture and carried out the experimentation. The usability evaluation consisted of 30 participants interacting with the autonomous robot in 30 min sessions. Then, 19 participants evaluated their perceptions of robot attributes of the Godspeed questionnaire by playing with the robot in 30 min sessions. The participants rated the Decision-making System with excellent usability (81.08 out of 100 points), perceiving the robot as intelligent (4.28 out of 5), animated (4.07 out of 5), and likable (4.16 out of 5). However, they also rated Mini as unsafe (security perceived as 3.15 out of 5), probably because users could not influence the robot’s decisions.

## Introduction

Social robots making autonomous decisions were devised to assist people in complex environments without a human controlling their behavior [14]. If the robot’s behavior adapts to the different situations and users, its performance is further improved [45]. As previous references state [12, 28, 41], generating dynamic behavior based on modeling natural phenomena has reported meaningful results in autonomous robots. More specifically, social robots intended to interact with humans require robust and adaptive decision-making to sustain the users’ attention correctly, keep them concentrated on the activity, and attain success [18]. Especially in long-term Human–Robot Interaction (HRI), maintaining the users’ engagement is essential for the robots to be accepted and employed in applications like entertainment, assistance, or cognitive stimulation therapies [16].

This paper presents an autonomous *Decision-making System (DMS)* for social robots that assist in cognitive stimulation and entertainment sessions. The primary novelties of the architecture are (i) it generates autonomous robot behavior drawing on human biology, (ii) it provides a system that deals with long-term interactions without the designers’ intervention, and (iii) it personalizes online the robot’s decisions to the users’ preferences, needs, and demands. These functions are addressed by incorporating into the decision loop the following features:

- Combine planned activities proactively proposed by the robot (using its biologically inspired behavior and an agenda) with the users’ demands. Thus, the system reacts to unexpected situations, dynamically changing the robot’s behavior to accomplish its goals.
- Provide a decision-making loop that continuously evaluates external stimuli perceived from the environment and internal signals (artificial needs) to generate adaptive robot behavior for long-lasting HRI.
- Obtain user information from HRI to adapt the robot’s decisions to the user features to produce a personalized interaction and action selection.

As we review in the following section, numerous DMS architectures have been developed in the last two decades. However, they lack in combining the previous features to endow social robots with long-term biologically inspired behavior. In this research line, we previously developed DMSs [25, 26] for social robots focused on reducing the robot’s artificial needs and personalized entertainment sessions. However, these architectures did not deal with unexpected situations experienced by the robot, were designed for short predefined HRIs, and partially considered the users’ petitions. Therefore, the DMS proposed in this paper pretends to overcome these limitations by mimicking human decision-making to improve the robot’s performance in social environments.

The system was evaluated in two experiments integrating the DMS in the Mini social robot [35]. These experiments assessed the robot’s usability, performance in terms of response times when managing the robot’s activities, activity exploration depending on the user proactivity level, and the errors committed while autonomously operating, and people’s perceptions of relevant robot attributes during HRI. In the first experiment, 30 participants evaluated the system *Usability* using the System Usability Scale (SUS) [8] by interacting with Mini controlled by the DMS. Furthermore, the performance metrics allow to analyze the usability ratings and verify the correct operation of the system. In the second experiment, we used the Godspeed questionnaire [6] to obtain the opinion of 19 participants towards the robot attributes *Anthropomorphism*, *Animacy*, *Likability*, *Intelligence*, and *Security* after interacting with Mini.

These experiments were conducted to evaluate three hypotheses that analyzed the users’ opinions and robot performance.

(**H 1**): The biologically inspired DMS drives to high robot usability.
(**H 2**): The performance metrics obtained during the usability test demonstrate the system’s good performance in terms of response times and errors yielded.
(**H 3**): The autonomous behavior of the robot under the control of the DMS makes their users perceive the robot as more human-like (anthropomorphic), animated, likable, intelligent, and secure (categories of the Godspeed questionnaire [6]).

This manuscript continues in “Related work” with a detailed review of DMSs based on biologically inspired concepts for autonomous social robots. “Mini social robot” presents the Mini social robot and its software architecture to understand how the DMS integrates into the robot. Then, “Decision-making system” presents our DMS, the cornerstone of this work, paying particular attention to its requirements, design, and implementation. “Evaluation” describes our experiments to validate the DMS and the results we obtained. Besides, we define the limitations of our system in real applications. Finally, “Conclusion” closes this manuscript with the main findings and future challenges for improving autonomous decision-making in social robots.

## Related work

In the late 1990s and early 2000s, the number of biologically inspired works describing how to endow social robots with autonomous behavior substantially grew [38, 40, 46]. These relevant neuroscience advances in the last years enable more complex and accurate models, integrating methods that emulate animal(human) behavior. Following, we explore the evolution of DMS for autonomous robots in the last 20 years.

Arkin et al. [2] developed a pioneering motivational framework for the autonomous behavior of the social robot Aibo. The model integrates aspects of animal biology, like emotions or physiological deficits, to shape the robot’s behavior. The system emulates biological functions in dogs to create an ethological framework where decision-making focuses on satisfying the robot’s physiological needs guaranteeing its survival. Two years later, Ávila-García and Cañamero [4] presented a biologically inspired model to modulate the motivational states of artificial agents. The regulation of the artificial variables draws on Schulkin’s [39] allostatic system, integrating the robot’s perceptions as essential modulators of the agent’s behavior. This work addresses autonomous behavior by acknowledging and adapting to unexpected environmental changes.

Konidaris and Barto [19] developed a motivational model for social robots that generates an autonomous robot behavior that maintains its artificial physiological needs in good condition. Thus, each deficit has a related behavior that satisfies the robot’s needs, so behavior selection depends on its highest biological deficit. Balkenius et al. [5] designed an action selection architecture based on the robot’s motivational states. Unlike the previous works, action selection does not focus on the agent’s physiological needs but on the motivational and affective states of the robot. The system emulates brain functions to represent essential processes involved in decision-making.

Some years later, Samani and Saadatian [37] presented an architecture based on affection for HRI. The model integrates physiological deficits to shape the robot’s behavior selection. The physiological state depends on the evolution of neuroendocrine responses that simulate biological functions in the agent. Then, physiology combines with psychology to define a state that allows the robot to make the most suitable decision. A distinctive factor regarding the previous works is the modeling of adaptive mechanisms towards the user considering the user-robot relationship level.

Lewis and Cañamero [21] designed a motivational module to control a mobile robot’s homeostatic physiological functions (hunger and thirst). The contribution of this work is the inclusion of an artificial hormone that regulates the robot’s pleasure in eating and drinking. Thus, the robot’s goal is to take actions that control its hunger and thirst, taking into account the effect of pleasure. Autonomous decision-making lies in an Action Selection Architecture (ASA) that maps the robot’s needs to specific appetitive (looking for food and drink) and consummatory behavior (eating and drinking). Like Lewis and Cañamero, Cervantes et al. [9] introduced a DMS for the intelligent autonomous behavior of social robots during HRI. The model shapes biological functions in human behavior to endow the robot with a robust physiological and psychological state that defines its action selection. The architecture integrates basic emotions as responses to environmental stimuli. Emotions influence the robot’s decision-making by activating specific behaviors. The model considers ethics in action selection to avoid behaviors that do not fit the context and situation.

Contemporaneously to the previous works, Adam et al. [1] developed CAIO, a Cognitive and Affective Interaction-Oriented framework for social robots in HRI. The framework integrates a DMS into a NAO robot endowing it with autonomous and emotional behavior. Decision-making depends on a deliberative layer that plans the robot’s behavior influenced by the robot’s emotions and a reactive layer that maps specific situations into reactive behavior to external stimuli. The robot’s behavior reacts to the robot’s emotions, leading to expressive communication with people. One year later, Lones et al. [22] designed a homeostatic robot controller. According to its dominant motivational states, the robot can exhibit autonomous and adaptive behavior. The robot’s internal state evolves with time depending on its behaviors and the influence of external stimuli. Thus, the robot aims to maintain its internal state while surviving in a dynamic world. This work’s most relevant novelty is hormones, which modulate the agent’s behavior simulating homeostatic mechanisms.

Recently, we [25] developed a biologically inspired DMS based on motivation. The system emulates biological functions like sleep or entertainment that influence the robot’s motivation (also influenced by stimuli) to control behavior during entertainment sessions with people. The system includes a model to learn from experience how to map each situation (state) to the robot’s behaviors (action). Although the robot exhibits autonomous behavior, the number of actions and situations the robot considers is small. This work does not consider planned activities and the user’s preferences.

**Fig. 1:**
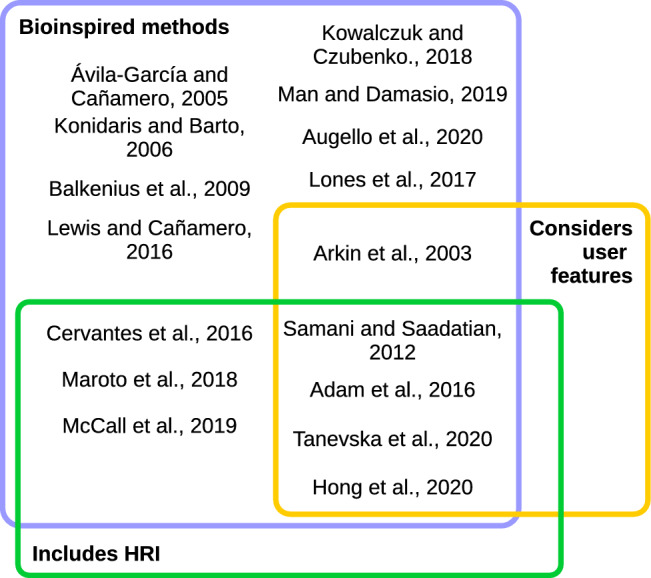
Classification of the related works regarding biologically inspired methods, user features to personalize the decisions and HRI methods

The Intelligent System of Decision-making (ISD) that Kowalczuk and Czubenko [20] carried out is one of the primary influences of our work since it considers the most important biological processes behind human behavior. The model, designed for social robots, considers the artificial physiological deficits of the agent as the central modulators of motivation. Besides, it shapes psychological functions like emotion and mood, influencing the robot’s decision-making and expressiveness. The system uses learning to consider the effects of each action on the robot’s internal state.

As reviewed above, the homeostatic and allostatic control in social robots has been deeply explored. For example, Man and Damasio [23] emulated a homeostatic model for social robots to exhibit autonomous and lively behavior. The model aims to simulate animal biological functions to allow robots to survive in dynamic environments by exhibiting adaptive mechanisms that improve the robot’s performance. Autonomous decision-making leads the robot to select the best action combination to overcome the adversities of the environment and survive.

McCall et al.[27] presented the LIDA architecture for the autonomous behavior of artificial agents. The software combines planned actions from the robot’s motivational state with emotional phenomena to select behavior. Behavior execution decomposes general high-level actions into simple motor commands. The model exchanges information with necessary external modules in the robotic software like perception, memory, or attention to improving the robot’s performance in HRIs and complex tasks. Besides, the robot’s expressiveness is modulated by emotions like happiness.

Like the previous works, Augello et al. [3] developed a motivational model to allow social robots to exhibit autonomous behavior. The architecture includes a human-like somatosensory system to produce physiological and psychological processes that urge behavior. Moreover, the decision-making learns which actions yield the best effects on the robot’s internal states and apply such information to improve the robot’s well-being in the long run.

In the same year, Tanevska et al. [43] introduced its software architecture for social robots. The robot’s decisions depend on a perception system that retrieves information from the environment and informs the DMS about the user’s state and interaction procedures. Then, a motor module controls the actuation devices by splitting general actions into specific motor commands. Finally, the adaptive system maximizes the robot’s well-being during HRI. The model includes an emotional system that considers happiness, sadness, and a neutral state for modulating the robot’s expressiveness. These emotions activate depending on the robot’s internal state and the emotions estimated by the user using visual information. Finally, Hong et al. [15] explored how affection influences the decision-making process in autonomous robots. The robot uses visual information to perceive the user’s emotions and adapt accordingly. The DMS combines deliberative processes with reactive behaviors in long-lasting interactions.

**Table 1:** Summary of the autonomous decision-making architectures considering the biological functions influencing decision-making, the user role, and HRI duration

Contribution	Use of physiological or psychological processes	Use of motivations	Use of affection	User role	HRI
Arkin et al. [2]	Simulates a dog physiology in AIBO robot	Dominant motivation urges behavior	Ekman’s 6 basic emotions [11]	Vague	No
Ávila-García and Cañamero [4]	Hormonal system modulates physiology and motivation	Dominant motivation defines behavior	Not included	None	No
Konidaris and Barto [19]	Physiological deficits are modeled as drives	Motivations reflect the robot’s drives	Not included	None	No
Balkenius et al. [5]	Models brain regions and their key processes	Motivations are inputs that affect the brain system, and action selection	Emotion modulate action selection	None	No
Samani and Saadatian [37]	Complex endocrine model controlling affection and physiological functions	Not included	Happiness, love, contentment influence decision-making	Decisions depend on HRI and user features	Short-lasting
Lewis and Cañamero [21]	Physiological system. Hunger and thirst. Pleasure hormone to modulate the robot’s pleasure to food and drink	Motivation compete to define the robot’s behavior by reflecting its needs	Not included	None	No
Cervantes et al. [9]	Physiological and pleasure systems	Motivations urge behavior to reduce deficits	Happiness, sadness, anger, fear, and surprise modulate action selection. Considers positive and negative moods	None	Medium-lasting
Adam et al. [1]	The robot’s state is defined by its physiological and mental state	Not included	Emotions are mental states with influence on decision-making	Robot behavior personalized to the user	Medium-lasting
Lones et al. [22]	Hormonal responses regulate the robot’s energy, health, and temperature	Motivational states urge behavior from the robot’s needs	Not included	None	No
Maroto et al. [25]	Entertainment and social need to control its fatigue	Three motivations that control playing, socialization, and fatigue	Not included	The user demands are not considered. The robot plays with the user to reduce its deficits	Long-lasting
Kowalczuk and Czubenko [20]	The mind model Physiological system (needs)	Motivation comes from the robot’s emotion, sub-emotions, needs, and mood defining its actions	The model considers emotion and sub-emotion in decision-making	None	Short-lasting
Man and Damasio [23]	Homeostatic model, mental states, and feelings	Motivation drives behavior from the robot’s internal state	Not included	None	No
McCall et al. [27]	Basic biological functions	Motivation is derived from the robot’s internal needs, stimuli, and cognitive factors	Emotion and mood	None	Short-lasting
Augello et al. [3]	Somatosensory system combined with needs, certainty, affiliation, and competence	Deliberative action selection based on motivations derived from the internal system	Vague mention of emotion in the decision loop	None	No
Tanevska et al. [43]	Physiological and psychological systems	Motivational states come from the robot’s needs. Action selection oriented to maximize well-being	Happiness, sadness, and neutral modulate the robot’s expressiveness	Robot decisions aim at providing good HRI	Long-lasting
Hong et al. [15]	A drive/desire system that represents the robot’s needs during the interaction	Not included	Emotions modulate action selection	User feedback affects the robot’s decisions	Short-lasting

As Fig. 1 shows and Table 1 summarizes, the literature highlights the influence and importance of biological functions, motivation, affection, and user features in the decision-making process. At a glance, it is possible to see that most of these works include motivation to urge behavior to reduce the robot’s deficits [2–5, 9, 19–23, 25, 27, 42]. Regarding the influence of affection on decisions, a few works consider emotions in the decision-making process [1, 3, 5, 9, 15, 20, 37], although a few of them [2, 9, 20, 27, 42] modulate behavior depending on the robot’s emotional state. Finally, only four works consider user preferences to adapt HRI, but all in short-lasting interactions [1, 15, 37, 42].

This paper fills the gap in developing autonomous social robots using a biologically inspired basis combined with personalized HRI for each user. Therefore, unlike the architectures presented above, we emphasize the user’s role in the loop for generating user-oriented adaptive mechanisms to personalize each user’s interaction because we combine the user’s demands to execute certain activities at will and we provide users with the possibility to tell the robot about when to execute planned activities using a virtual calendar. Since we devised to assist people, in our framework, the user is the primary source of information that the robot uses to adapt its behavior while executing autonomous tasks.

## Mini social robot

**Fig. 2:**
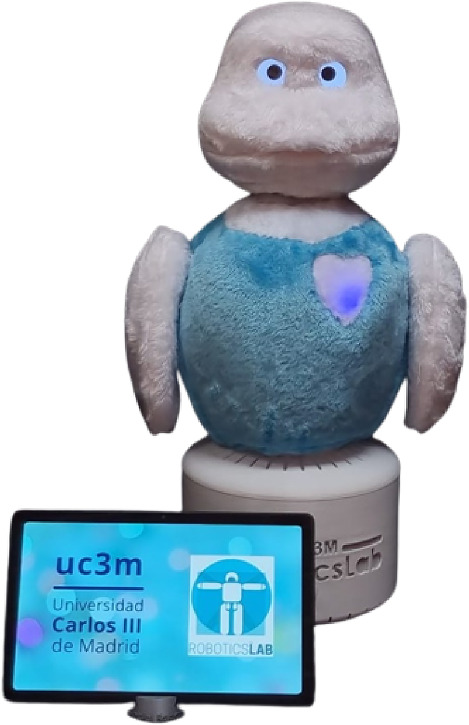
The Mini social robot, the platform used to integrate the DMS, is dedicated to alleviating seniors’ cognitive impairment by executing a broad repertoire of activities

Mini [35] is a social robot conceived to work on cognitive stimulation therapies and entertainment in face-to-face HRIs. As Fig. 2 shows, Mini is a desktop platform with a touch screen to display information to the user. The robot verbally communicates with people using text-to-speech software and an automatic speech recognition system that manages the conversation (voice). Its foamy case has three touch sensors to perceive caresses and hits (touch). Mini has 5 motors in the hip, arms, neck, and head and RGB LEDs to express its state with different colors. The robot has a 3D camera with a depth sensor to perceive the user and other environmental stimuli (vision).

### Software architecture

**Fig. 3:**
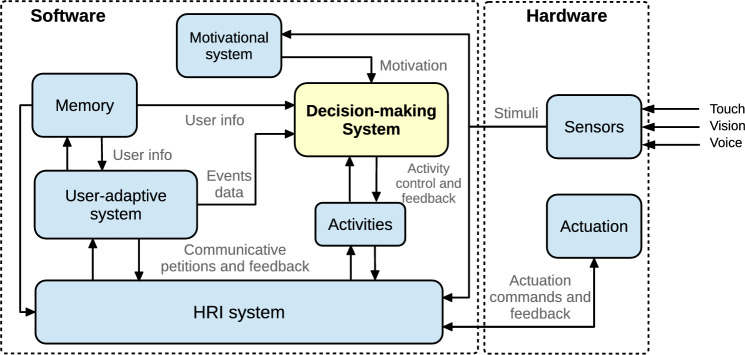
The software architecture of the Mini social robot highlights the DMS situation and its communications (in yellow)

This section describes the software architecture where the DMS has been integrated, as shown in Fig. 3. Their aggregate operation was specifically designed for the autonomous control of the Mini social robot. We emphasize those modules related to the DMS and provide them with meaningful information to make decisions.

#### Memory

The software architecture of our robots has a Memory module that stores information about the robot and the users that interact with it. In this module, each user has a unique personal profile that contains their features (e.g., name or age) and preferences towards the robot’s activities. This module communicates with the other modules in the architecture that adapt the interaction using the user information, like the DMS. Among these communications, the principal information exchanges occur with the User–adaptive system and the HRI system. On the one hand, the HRI system manages the interaction, so the robot must obtain the user’s features to adapt to every specific user. On the other hand, the Memory exchanges information with the User–profiling system since this module handles the retrieval of new user information and informs the Memory to load the user’s profile.

#### Motivational system

The *Motivational system* [25] controls the artificial biological processes emulated in the robot to generate a biologically inspired and lively behavior during long-lasting periods. It receives information about the stimuli the robot perceives and makes the biologically inspired processes evolve with time, simulating physiological and psychological functions like sleeping or emotion. The deficits in these biological processes due to the stimuli the robot perceives and their daily evolution define the robot’s motivation. Motivations can be defined as states that urge behavior, leading to behavior selection. Since many motivations can be simultaneously active because the robot can present many deficits, we describe the dominant motivation as the motivation with the highest level of intensity. The dominant motivation is attached to a specific behavior, and its goal is to restore the robot’s internal state and reduce the deficit with the highest intensity level. Consequently, the Motivational model informs the DMS about the behavior to execute to maintain the biological processes of the robot in good condition.

#### User-adaptive system

The User-adaptive system aims at adapting the robot interaction to the user. It is connected to the robot’s *Memory* to exchange the user information. This module communicates with the HRI system to know the user the robot perceives and loads its information to personalize the interaction. During the interaction and the execution of activities, this system uses the user’s feedback to represent how much the user likes the activities or the limitations they present during the interaction.

A paramount module inside the User-adaptive system is the *agenda*. This module stores information about activities that each user has to execute on specific dates and times. The agenda considers two types of activities: events and reminders. On the one hand, events are activities the user has to execute at a specific time, such as performing cognitive stimulation exercises. On the other hand, reminders are short communications that the robot tells the user at a particular time, like an appointment with the doctor. The events and reminders are stored in the robot’s memory module, and every time the robot perceives a different user, the user’s schedule is loaded into the agenda. Then, when the event/reminder is about to start, the information is sent to the DMS for its execution.

#### HRI system

The HRI system [13] is the manager inside the robot architectures dedicated to controlling and facilitating HRI. In our approach, the HRI system is integrated by three subsystems that exchange information among themselves and other modules in the architecture to interact with the user successfully. The subsystems that integrate the *HRI system* are:

- **Perception manager:** This module receives information from the sensors of the robot and translates this information into a general message that the rest of the system can easily interpret.
- **HRI manager:** This module acts at an intermediate level between the *Perception manager* and the Expression manager. On the one hand, it receives information from the Perception manager about what the robot perceives and conveys this information to the modules that request it. On the other hand, it receives data from the other modules in the architecture about how to interact, sending appropriate commands to the Expression manager about controlling expressiveness.
- **Expression manager:** The Expression manager controls the robot’s actuation and expressiveness. This module receives general abstract expressions that the robot has to perform. Then, it manages their execution, avoiding conflicts between petitions and decoupling these available instructions into individual commands for each actuator.

#### Activities

The activities are the functionalities that the robot can execute. They are essential to exhibit autonomous and lively behavior. They comprise actions like sleeping or informing about important events. The robot activities can be continuous or discrete. On the one hand, continuous activities are always working, covering general robot functionalities such as managing communication with the user. On the other hand, discrete activities become active under the control of the DMS and entail the execution of punctual functionalities like playing a game. The discrete activities are stopped by default, but can be started, paused, resumed, and canceled depending on the situation the robot is experiencing and the user will.

**Table 2:** DMS requirements and their design proposals

Requirement	Our design proposal
The DMS requires computational resources to control the robot’s behavior. Since it evaluates much information from sensors and other modules making decisions to manage the execution of different activities, the system needs a powerful computer with a distributed framework to develop the proposed software architecture	The software architecture and DMS runs on a Intel Core $i7-7700K$ with the Asus *Z*170*I* Pro motherboard and 8GB RAM. The software builds upon Ubuntu 20.04 and a customized setup of the Robotic Operating System for the communications between modules
The user is always a priority for the robot, so whenever it detects a user ready to interact, the behavior must be adapted to meet its needs	The robot uses visual information from the perception system to recognize the user, load its information if it exists, extend it with HRI, and adapt the interaction
The robot’s behavior must be user-adapted. It must include mechanisms to obtain user information, store it, and adapt the interaction	The User-adaptive system provides the DMS with information about the user. Using this information, we propose to personalize HRI
The robot must sustain the users’ engagement by keeping them active and entertained. Thus, the users are focused on the robot and execute the proposed task successfully	The system works uninterruptedly, evaluating the external information and generating appropriate behavior to engage users
The robot must be able to cancel, pause, and resume an ongoing activity, improving the system reactivity and dynamics and allowing the user to execute the activities they want	We propose that by touching Mini’s right shoulder, users indicate their intention to change the activity (other robots can implement another mechanism). To make sure the user touched the shoulder intentionally, the robot asks the user to continue or stop the activity
The robot should detect possible limitations in how users interact (e.g., not using the touch screen correctly) and adapt the interaction to overcome these situations	We propose a three–mode DMS: (1) a fully proactive mode where the robot makes autonomous decisions, (2) a reactive mode where the robot lets the user select the activity, and (3) a hybrid mode that combines both previous cases. The operating mode depends on the *user’s proactivity*, which defines the user’s initiative to start interactions
The DMS must maintain a lively robot behavior giving the sensation of being alive and ready to interact	We propose that when no user is detected, the Motivational model sets a biologically inspired behavior emulating humans
The robot should store planned user events and reminders and execute them on specific dates and times	0ur architecture includes an agenda that stores events and reminders that the robot must execute and notify the user. The agenda receives information from the robot’s Memory about the activities and reminders of each particular user. When the starting time approaches, the DMS executes the activity
Since social robots are for entertainment activities such as playing multimedia or telling the last news, and each user has their preferences, they must be aware of them and personalize the proposed activities	We propose two mechanisms to personalize activity selection: (1) The robot predicts the users’ preferences based on their features [26]. If these features are not available, the prediction is random. (2) After executing an activity, the robot asks the users whether they like it to adapt to new interactions

The activities are intrinsically related to the DMS since the final goal of our system is to manage their execution appropriately. Besides, since all activities follow the same format and are devised to control actuation, they provide the following benefits.

- The activity control can be generalized as all activities are equally controlled and designed. This modeling method improves our system’s scalability, modularity, and flexibility since developers can easily integrate their activities into the robot.
- Improves the maneuverability allowing the robot to exhibit adaptive and more natural behavior since the DMS can dynamically change the activities.
- Simplifies incorporating new activities into the system, as developers have a well-defined method for increasing the robot’s functionalities.

The current activities of our robot aim at entertaining the user, performing cognitive stimulation, or exhibiting a biologically inspired behavior. At present, Mini can perform the following activities:

- **Sleep:** The robot simulates it is sleeping.
- **Wait:** The robot waits for new upcoming events without doing any specific action.
- **Play multimedia content:** The robot plays multimedia content on the touch screen (e.g., music, audiobooks, photos, videos, and films).
- **Tell the last news:** The robot looks for the last news on the internet and tells them to the user.
- **Tell the weather forecast:** The robot looks for the weather forecast on the internet and tells it to the user.
- **Play a quiz game:** The robot plays with the user a quiz game. The robot asks the user about different topics and provides four possible answers. The user has to select the correct answer.
- **Perform cognitive stimulation:** The robot starts a predefined session containing cognitive stimulation exercises to train memory, perception, and other skills.
- **Talk with the user:** The robot has a predefined list of dialogues to maintain the user engaged in the interaction.
- **Dance:** In this activity, the robot plays a random song and starts dancing.
- **Bingo:** The user plays the famous Bingo game with the robot.
- **Jokes:** The robot says three Spanish jokes to make the user laugh.
- **Sayings:** The robot tells the user Spanish sayings.
- **Presentation:** The robot presents itself and its main capabilities during a short speech.
- **Instructions:** The robot shows the user how to use its functionalities.
- **Tablet menus:** This activity controls the menus displayed on the touch screen, allowing the user to navigate between them and select the activity they prefer. The user makes the selection using the touch screen or voice commands.
- **Notifications and communications:** The robot informs the user about upcoming events or reminders.

## Decision-making system

This section presents the DMS proposed in this paper to control the autonomous behavior of social robots. First, we highlight the initial requisites that drove the system’s design. Then, we describe the system operation enumerating its principal functions to produce autonomous behavior combining planned activities with responses to unexpected events.

### Requirements and design

Before developing our DMS, we thought about the features it should have to control the robot during cognitive stimulation and entertainment sessions. Consequently, we defined a set of initial system requirements and their proposed solutions, as Table  2 shows.

### Operation

**Fig. 4:**
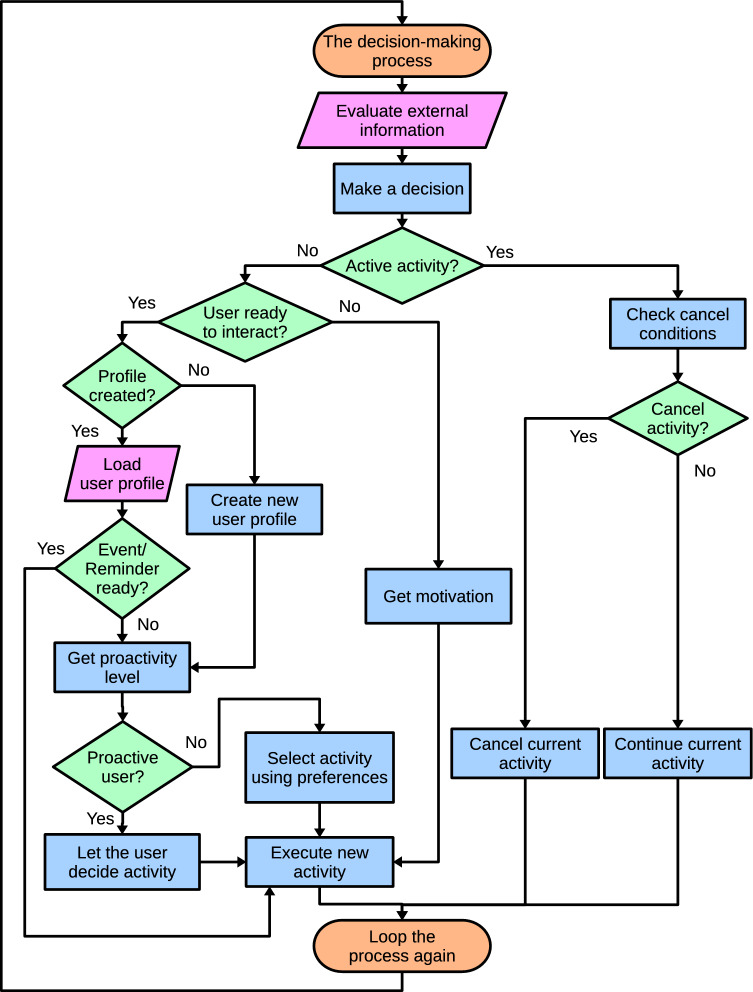
The decision-making loop manages the robot’s decisions. The adaptive mechanisms take place at different points of the decision-making loop. First, the user’s proactivity indicates if the user is more likely to select the activities or requires robot assistance to start the interaction. Second, user preferences suggest their favorite activities according to their features. Finally, the activities and reminders the robot executes/notifies are unique for each user, personalizing the activities performed

After defining the system requirements and their design solution, this section addresses the DMS’s operation and how it controls the activities. As the flow chart in Fig. 4 shows, the DMS evaluates the information from the other modules and makes a decision. If the information of a module is missing, that module is not considered in the decision. The evaluation takes place every 0.5 second (the frequency with which the other modules send the information to the DMS) and depends on whether there is an ongoing activity or the robot has to select a new behavior.

**Fig. 5:**
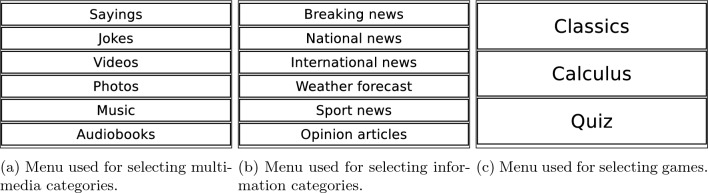
Tablet menus are shown to the users on the touch screen so they can select the activity they prefer

The decision loop describing the evaluation of the DMS inputs and the behavior selection is as follows.

1. In every iteration, the DMS evaluates the information received from all the modules affecting the robot’s decisions.
2. The first information that the DMS checks is whether there is an ongoing behavior. If the robot is executing a behavior, the DMS evaluates the information received from the other modules to decide if the behavior has to be canceled. There are four possible reasons to cancel an ongoing behavior. They are evaluated in the following order:
  If the user is not interacting with the robot and behaves according to its motivational state, the current behavior changes if the dominant motivation changes.
  If the user requests to change an activity, the robot fulfills the request.
  If the agenda informs about a new upcoming event/ reminder with high priority.
  If an activity returns an error or indicates that another activity must be executed (e.g., activating a game after asking the user what to do).
3. If the robot is not executing any behavior, the DMS has to make a new decision and checks, using the perception information, if a user is ready to interact with the robot.
4. If there is not a user ready to interact with the robot, the DMS selects the following behavior using the information provided by the Motivational system. This module mimics biological processes in the robot to improve its liveliness and expressiveness. The Motivational system directly provides the most appropriate behavior according to the robot’s internal state, reflected through the dominant motivational state (e.g., suggesting to sleep when the dominant motivation is resting because the robot is tired).
5. If a user is ready to interact, the memory manages the user’s profile. If the user profile is already created, the DMS loads it and uses the information to adapt the interaction. Otherwise, if the profile is not in the robot’s memory, the robot creates an empty one that is filled during the interaction.
6. At this point, the DMS verifies if the agenda has an upcoming event/reminder. If a new event or reminder is ready, the robot executes it. Otherwise, the robot retrieves the user’s proactivity level to work in the following operation modes.
  If the user proactivity is high, the robot typically gives the initiative to the users so they can select the activity they prefer using the menus shown in Fig. 5 or saying the activity they want to execute (Mini can understand speech). These menus are displayed on the screen and are organized in different categories and levels (see [26]).
  If the user proactivity is moderate, the DMS will work on a hybrid mode that will combine proactive autonomous decisions with ceding the initiative to the user. The user proactivity level does not implicate using the same operation mode for a specific user but assigns a higher probability to one of the modes. This mode also becomes active if the robot cannot retrieve the proactivity level.
  If the user’s proactivity is low, the robot will make an autonomous decision using the user’s preferences.
7. After executing an activity, the robot usually asks the users how much they liked it, updating the user profile with their preferred activities. This question is only sometimes presented since it depends on a probability value ($50\%$ chance to be asked).
8. Once in a while, to avoid fatiguing the user with repetitive activities, the robot incorporates general questions into the interaction to improve the communication between both agents and retrieve user information for updating the user profile.
9. After all these steps, the cycle repeats.

This loop repeats, allowing the robot to select the proper behavior while dynamically evaluating the received inputs. Note that the behavior selection process combines a motivational biologically inspired behavior with adapting to the user’s features to maintain engagement by personalizing activity selection.

**Fig. 6:**
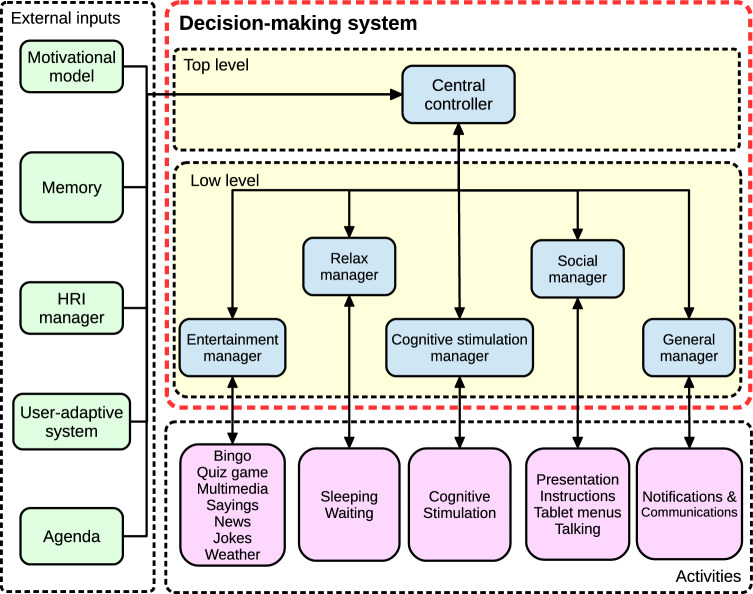
The DMS (red) is organized into two levels, the Central controller and the Managers. The Central controller receives the information from other architecture modules and decides which activity to execute. Then, the corresponding Manager handles the execution of the activity

### Implementation

The DMS consists of two hierarchical levels that exchange information using the Robot Operating System [32], a framework that allows developers to build robotic applications by providing software libraries that simplifies the integration of each module in the software system. As Fig. 6 shows, these two levels are the *Central controller* and the *Manager*. The Central controller manages the information received by the rest of the modules, processes it, and makes the first decision about how the robot should behave. Then, the Central controller activates one of the five Managers located one level below in the architecture. The Managers have two purposes. They alleviate the Central controller’s computational charge and control the robot’s activities using bidirectional synchronous communications.

The Manager level has five separate Managers, each controlling a different group of activities. As we detail below, the classification of which activities are managed by each Manager depends on their theme. Sometimes, a Manager can make a partial decision about the activities’ details (e.g., deciding the type of entertainment activity proposed to a specific user). Below, we take a closer look at the functionality of the Central controller, the Managers, and how both coordinate to control activity execution.

#### Central controller

The Central controller is the main module of the DMS since it makes the most critical decisions about how the robot has to behave. As mentioned above, this module receives the information from the external modules of the DMS, evaluates all the information, and follows the flow chart depicted in Fig. 4. Once the Central controller has decided, it activates the corresponding Manager to control the robot’s activity. The Central controller and each Manager continuously exchange information about their status and the status of the active activity, evaluate all possible situations, and act accordingly. The exchange of information is bidirectional and synchronous to strengthen the system’s operation. This fact means that every time the DMS sends a command to a Manager, the Manager has to respond, updating its status and the status of the controlling activity.

#### Manager

The Manager level contains five managers: *Entertainment*, *Social*, *Relax*, *Cognitive stimulation*, and *General*. The Central controller manages them, and each Manager controls a group of activities, as Fig. 6 shows. By default, all Managers are deactivated and only become active under the petition of the Central controller. Once active, the Manager keeps track of the execution of the robot activity demanded by the Central controller.

The following list describes the functionality of each Manager in our architecture.

- **Entertainment manager:** Controls the robot’s entertainment activities. It includes a predictive system [26] that estimates each user’s preferences, personalizing the activity selection during entertainment sessions. This Manager can make partial decisions about which particular entertainment activity the robot has to execute, adapting to the user.
- **Social manager:** Controls all the activities that involve talking with the user (conversation).
- **Relax manager:** Controls the activities of sleeping and waiting for upcoming events, denoted as relaxing activities.
- **Cognitive stimulation manager:** Controls the activities oriented to conducting cognitive stimulation therapies with the user. These sessions are generated as described in [36].
- **General manager:** Manage short notifications that the robot has to make to the user, like reminding appointments.

Our design allows the robot to execute one activity at once, but when the robot has to make a short communication to the user (e.g., a reminder), it is possible to pause the current activity, make the announcement, and resume the previous activity again. Thus, we allow the robot to alternate two activities during short periods. In the DMS, the General manager handles short communications while the other activity is controlled by the Entertainment, Social, Relax, or Cognitive stimulation managers.

**Table 3:** Details of the experiments carried out to evaluate the performance of the DMS

Experiment feature	Usability functions used	User perceptions
N of participants	30	19
Age range	18 to 52	18 to 56
Genre distribution	11 male, 12 female	7 male, 12 female
Participants’ demographics	Spaniards University members	Spaniards General audience
Session duration	30 min	30 min
Number of sessions	1	1
Evaluation method	SUS [8]	Godspeed [6]

## Evaluation

This section describes the scenario, experimental setup, evaluation method, and the results of the DMS presented in this manuscript. First, we describe the HRI scenario where both experiments were carried out.

The evaluation took place through two studies with different participants using our Mini social robot during HRI. The details of the experiments are in Table 3. The robot exhibited autonomous behavior controlled by the DMS during the interaction. The first experiment measured the systems’ usability, while the second evaluated the users’ perception of specific robot attributes.

A video^1^ recording about how our DMS works in HRI sessions complements this paper. The video shows the DMS dynamics following the steps enumerated in “Decision-making system”. The video also emphasizes the three operation modes depending on the user’s proactivity, how the user cancels an ongoing activity, and the control of activities.

**Fig. 7:**
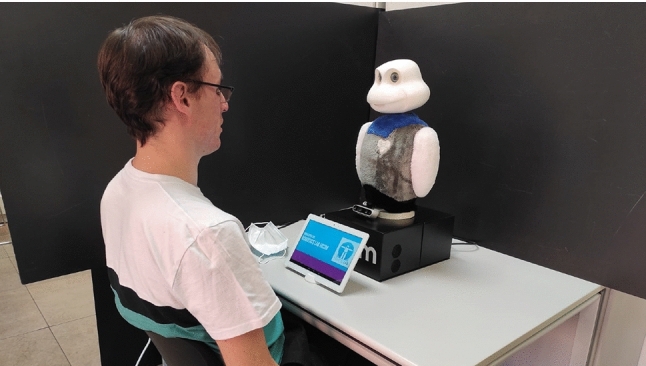
Interaction scenario used to evaluate the DMS in terms of usability and user perceptions. The user is seated in front of the robot to perform the sessions guided by the robot

### Scenario

The Mini social robot is a desktop robot that requires the user to be seated in front of it to interact together successfully. Due to this constraint, the interaction scenarios used for evaluating the usability and user perceptions followed the same pattern and similar dynamics.

The scenario, shown in Fig. 7, consisted of the user sitting on a chair situated in front of the robot. Once in front, the participants consented to participate in the experiments. Next, they were told how to cancel an activity, that the activities are displayed on the touch screen, the duration of the session (30 min), to press *Start* on the screen when ready, and to pay attention to the robot’s indications. Following, “Experimental setup” and “Experimental setup” provide information about each experiment, the number of participants, and the dynamics of the session.

### Experiment 1: usability and performance metrics

The following sections describe the experimental setup and show the results of the usability ratings provided by the participants and the performance metrics obtained during the session.

#### Experimental setup

In the first experiment, 30 participants (11 men, 19 female), all Spaniards aged 18–52, interacted with the Mini social robot during individual interactions that lasted around 30 min. The participants receive information about how to cancel an activity, that the activities are displayed on the touch screen, the duration of the session (30 min), to press *Start* on the screen when ready, and to pay attention to the robot’s indications before interacting with the robot; none had previously interacted with a social robot. However, some of them watched videos about the Mini social robot and have some expertise in robotics. The interaction process started with the user seated in front of the robot in an empty room. After pressing Start, the robot asked the user general questions to initiate the interaction (e.g., What is your name?, How are you?). The robot took the questions from a poll without repetition. Then, after completing 4–5 general questions, the participants executed 4–6 different entertainment activities until the session finished. Activity selection was carried out autonomously by the robot.

During the session, in half of the activities executed, Mini let the participant select the activity they preferred using the touch screen menus shown in Fig. 5. The robot autonomously chose the activity the other half of the time. In each session, each participant completed at least five activities from the repertoire indicated in Sect. “Activities”. Finally, after completing the activities, all participants completed a survey to rate the system’s usability. Filling out the usability survey lasted around 3 min of the session time.

We used the Spanish version [44] of the SUS questionnaire [8] for measuring the usability of the system (in this case, the robot controlled by our DMS). The questionnaire consists of 10 questions ratings using a 5-point Likert scale. The questions measure how people perceive DMS usability using the Mini social robot. The DMS’s usability is on a 0 to 100 scale, where higher ratings indicate excellent system usability. Although different interpretations can be made from the usability value obtained from the questionnaire, we use the one provided in [47] that maps each usability value in the 0-100 scale into a quality term. If the usability value is below 60 units, the usability is considered *low*; between 60 and 80 units, the usability is regarded as *moderate*; above 80 units, the system’s usability is *excellent*.

#### Usability results

**Fig. 8:**
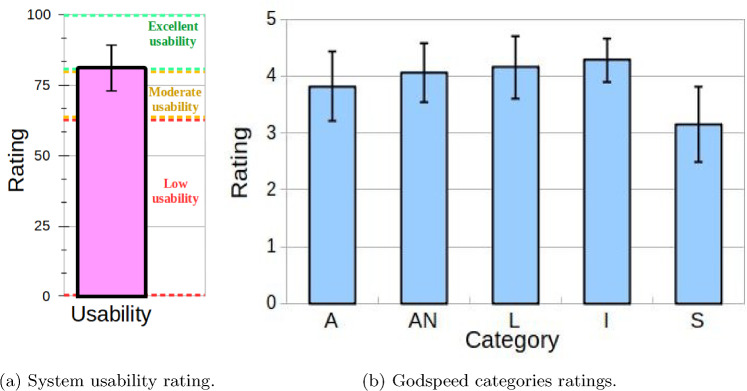
Ratings provided to the a) DMS usability using the SUS scale and b) robot Anthropomorphism (A), Animacy (AN), Likability (L), Intelligence (I), and Security (S) perceived by the participants using the Godspeed questionnaire

The first experiment’s results for measuring the system’s usability using the SUS questionnaire [8] provided valuable outcomes. The 30 participants of this study rated the overall usability of our approach with mean $\mu =81.08$ and standard deviation of $\sigma =11.46$, as Fig. 8a shows. According to [8], a system’s usability can be considered low if it is below 60 units, good between 60 and 80 units, and excellent if above 80 units. Considering this classification, our DMS’s usability is excellent since the average usability provided by the participants was 81.08.

This result supports our Hypothesis 1 (**H1**) stated in Sect. “Introduction” about obtaining a positive system usability value for our robot controlled by our DMS. We interpret excellent system usability as an indicator showing that the users found our system easy to use. Since our architecture is designed to assist people in cognitive, affective, and entertainment sessions, obtaining easy-to-use systems is critical for the users to correctly fulfill their tasks with the robot and engage with it.

#### Performance metrics

During the usability test described above, the DMS logged information about the interaction dynamics and its performance to validate Hypothesis 2 (**H2**). Table 4 shows these metrics classified into three groups. The first group, called *performance metrics*, provides information about the DMS performance, like the time to load each activity. The second group includes information about the participants’ *use of the DMS functions* like canceling an activity by touching the robot’s shoulder. Finally, the last group contains information about the user, and DMS *failures* encountered during the session. The performance and use of the DMS functions are expressed by their main and standard deviation per session. The failures during the interaction are defined as the total number considering all the sessions. These metrics, whose results are discussed in Sect. “Decision-making System”, are presented to show the response of the DMS and the participants’ actions during the session. As we can see at a glance, the robot balanced autonomous decisions by letting the user select the activity while making few errors (only 9 in 30 sessions).

**Table 4:** Performance metrics of the DMS and the user obtained during the usability tests

Metric type	Metric	Values
Performance	Questions per session	3.78 (0.56)
Performance	Activities per session	4.76 (0.42)
Performance	Activity average loading time (s)	3.78 (0.25)
Performance	Activity execution average time (s)	255.91 (62.89)
Performance	Average time to change activity (s)	43.12 (3.21)
DMS functions	Robot autonomous decisions	2.30 (0.26)
DMS functions	User activities selection	2.46 (0.18)
DMS functions	Cancel ongoing activity	0.53 (0.09)
Failures	Activity not loaded	2
Failures	Speech recognition errors	5
Failures	User not answering	0
Failures	Other DMS errors	1

### Experiment 2: User perception of the robot

The following sections describe the experimental setup and shows the results of the evaluation carried out to measure the users’ perceptions towards specific robot attributes.

#### Experimental setup

In this second experiment, 19 participants (7 male, 12 female), all Spaniards aged 18 to 56, assessed some robot attributes during entertainment sessions while making autonomous decisions. This second experiment was carried out after measuring the system’s usability in *Experiment 1* and assuring that robot users find our system easy to use. No participant in this study took part in the usability one. Besides, they had no robotics expertise and had never been face-to-face with a social robot. The participants received information about how to cancel an activity, that the activities are displayed on the touch screen, the duration of the session (30 min), to press *Start* on the screen when ready, and to pay attention to the robot’s indications before interacting with the robot.

This second study aimed to obtain the users’ perception of different robot attributes while making autonomous decisions controlled by the DMS. Again, the interaction started with the user seated in front of the robot in an empty room. Each participant individually interacted with the robot in a 30-minute session, performing three entertainment activities selected by the robot. The activity selection was performed using the preferences generated by the *Preference Learning* system described in Sect. “Decision-making system” and presented in [26]. The preferences prediction was generated using the user features and Label Ranking, a preference predictor that produces a ranking of each user’s preferences from a dataset containing the characteristics and preferences of similar users. To avoid biasing the experiment, all participants completed a personal questionnaire before the session for the robot to predict their preferred activities. Completing the initial personal questionnaire took each participant around 10 minutes of the session since it contained 30 questions. After completing the three activities, the participants filled out a survey rating robot attributes. The filling of the questionnaire took, on average, around 5 minutes of the session time.

We used the Spanish version^2^ of the Godspeed questionnaire [6] that measures the users’ perception towards the robot’s attributes *Anthropomorphism*, *Animacy*, *Likeability*, *Intelligence*, and *Security*. We used this questionnaire because it is widely used in social robotics and covers the features we wanted to assess from our robot being controlled by the DMS. Each category measured in the questionnaire contains 3 to 6 sub-attributes that the study participant has to rate using a 5-point Likert scale. Then, the average rating of the sub-attributes is computed to produce an overall rating that defines each category. Although the interpretation of the Godspeed questionnaire has been previously addressed [24, 47], there is not a unified criterion for discerning what is considered a high or low value for each category. However, these studies suggest those mean ratings above 3.5 point out of 5 in each category can be considered positive.

#### Godspeed results

Figure 8b shows the ratings provided by the 19 participants that carried out this study. The participants rated the robot’s Anthropomorphism with mean $\mu =3.81$ and standard deviation $\sigma =0.60$, Animacy with $\mu =4.07, \sigma =0.52$, Likability with $\mu =4.16, \sigma =0.55$, the Intelligence perceived with $\mu =4.28, \sigma =0.39$, and the Security perceived with $\mu =3.15, \sigma =0.66$.

According to the interpretation of the Godspeed ratings stated in [24, 47], the participants rated the Animacy, Likability, and Intelligence perceived as positive. However, the category Security perceived was moderately negative since its value is around three units. Finally, the participants rated the robot Anthropomorphism as relatively positive (subtly below four units). These outcomes suggest that our Hypothesis 2 (**H2**) stated in Sect. “Introduction” is only partially accomplished. On the one hand, the results for Animacy, Likability, and Intelligence suggest that people perceive the robot behavior as animated, likable, and with some degree of intelligence. These results show positive impressions from the participants about the robot’s actions and activity selection.

Nevertheless, the moderate rating obtained for the Security perceived category suggests that participants perceive the robot as an insecure machine. A possible explanation for this rating might be that participants saw the autonomous robot behavior as something they cannot influence or modify. Finally, the results for the Anthropomorphism category might indicate that participants see the robot as quite human-like, although the rating of this category does not attain values to be fully considered positive.

**Table 5:** Usability results of this work compared to similar studies in autonomous social robots

Paper	Pax	Usability rating
This work	30	81.04
Di Nuovo et al. [10]	36	76.40
Olde Keizer et al. [29]	21	60.50
Broadbent et al. [7]	40	78.24
Zou et al. [48]	15	$>70$

**Table 6:** Robot perception in other studies using the Godspeed questionnaire. Anthropomorphism (A), Animacy (AN), Likability (L), Intelligence (I), and Security (S)

Paper	Pax	Godspeed results
This work	19	A = 3.81; AN = 4.07; L = 4.16; I = 4.28; S = 3.15
Saldien et al. [34]	162	A = 4; AN = 3.6; L = 4.3; I = 3.6; S = 3.7
Petisca et al. [30]	30	A = 3.18; AN = 3.31; L = 3.49; I = 4.08; S = −
Khan and Germak [17]	48	A = 2.90; AN = 3.92; L = 4.28; I = 3.95; S = 4.38
Piasek and Wieczorowska [31]	5	A = 3.08; AN = 3.13; L = 5; I = 4.12; S = 4.27
Roesler et al. [33]	200	A = 1.80; AN = 2.20; L = 3.60; I = 3.40; S = 3.94

### Results discussion

The system evaluation was carried out in two HRI experiments directed to different groups of participants. The participants in the first experiment, which aimed at measuring the system’s usability, provided valuable ratings. On average, the system usability was above 80 units, considered excellent by the interpreters [47] of the SUS scale [8]. Since people with and without expertise in robotics participated in the experiment, we can conclude that generally, they found our system easy to use and understand, especially during the robot’s decision-making and the execution of the robot’s entertainment activities. Besides, similar usability results with autonomous social robots (see Table  5) have reported subtly lower usability ratings (around 75 points out of 100) than our work. The positive outcomes of our evaluation might be related to the performance metrics in Table 4. As we can see, the system only yielded 8 errors during the 30 sessions, most of them due to not recognizing the users’ speech during the activities (5), not loading an activity that was not correctly defined (2), or reporting other errors in the communications between modules (1 message lost). As the performance metrics demonstrate, the system allows dynamic sessions with a balanced robot and user decisions, allowing users to cancel an activity if they do not like it or prefer to execute a different one.

From the computational point of view, the DMS performance reported low response times to react to unexpected situations. As the performance metrics in Table 4 show, the average time to load a new activity is around 4 s, and the average time to cancel an ongoing activity due to a user petition is around 45 s. These response times were obtained using a powerful embedded computer as indicated in the system requirements (see Table  2). These responses might be subtly affected by the processor where the software runs. However, we believe that these minor delays might not have an important impact on the system’s performance and users’ perceptions.

The second experiment evaluated how people perceive some robot attributes while autonomously making decisions in entertainment sessions. Using the Godspeed questionnaire [6], we measured the participants’ perception of the robot’s Anthropomorphism, Animacy, Likability, Intelligence, and Security. On the one hand, people perceive the robot as animated, intelligent, and likable, as these categories’ rating was above 3.5 points from 5, and we consider this rating as positive based on [24, 47]. However, the rating in the Anthropomorphism category was almost half a point below Animacy, Likeability, and Intelligence, maybe because our robot looks more like a toy or character than a human. On the other hand, the rating of the *Security* category was almost one point lower than the others (3 from 5), probably indicating that people without expertise in robotics perceive the robot as unsafe on some occasions or as threatening. Another possible cause of this issue is the lack of control that the participants may perceive when interacting with an autonomous robot. Since people are habituated to using fully teleoperated machines, they probably perceive autonomous social robots are unsafe because they do not know what they will do during the interactions.

Compared to similar studies that used the Godspeed questionnaire with autonomous social robots (see Table 6, our work yields similar values for the categories Anthropomorphism, Animacy, Likability, and Intelligence. However, the category Security reports significantly lower results compared to these studies. A possible cause might be that their evaluations were conducted using videos (providing many participants) and not real interactions. This means that people may feel more secure when assessing robot features using recordings and not participating in real HRI.

### Limitations

The architecture presented in this paper has some limitations that should be considered when deploying the system in real scenarios. Following, we enumerate them and provide possible solutions that should be addressed in the future.

1. The primary limitation of implementing the system in real application is that not all the features of the DMS have been evaluated, so some capabilities might not be the best option design for HRI. We believe that further experiments are necessary where the different functions of the DMS are evaluated individually. However, this is tedious due to the many different features integrated into the architecture. Consequently, in this study, we opted to evaluate the system in terms of usability and users’ perceptions of the robot.
2. The DMS includes different features that depend on the device’s hardware features where it is integrated. Therefore, if the system is to be integrated into another robot that lacks sensor or actuation devices, the performance could be improved (e.g., with a camera, the robot can recognize the user, and dynamic adaption is possible). Contrarily, the DMS will benefit from a robot with better hardware capabilities since the action repertoire can be expanded and adapted.
3. Related to the previous point, another significant limitation is the action repertoire of the robot. In this paper, we have presented the activities that Mini can execute. The DMS aims at controlling these activities, so their design should follow the methodology presented in this contribution.
4. The DMS operates under three different modes that depend on the user’s proactivity. This parameter regulates when the robot makes an autonomous decision (low user proactivity) and when the robot lets the user decide on the following activity (high user proactivity). In the evaluation presented in this manuscript, we set moderate proactivity for all users, so more experiments are necessary to analyze the real impact of this parameter on the users’ opinion about the DMS operation.
5. As we described in Sect. “Requirements and design”, we specified some requirements before designing the system. Among them, we defined the computational needs to run such a complex and modular system. We believe that current computers have enough power to run our DMS, but if it is combined with new features like Machine Learning, powerful computers might be needed.

## Conclusion

Autonomous social robots expand the possibilities of robotic systems in social environments. As discussed earlier in this paper, robots in HRI scenarios require adaptive mechanisms to personalize the interaction and facilitate the use of the system to engage users and attain their acceptance. The DMS presented in this paper aims at providing such functionalities by controlling the execution of planned activities, regulating the robot’s biologically inspired processes, and providing adaptive, personalized behavior to the users.

The previous studies validate the operation of the architecture in real HRIs. However, the DMS presented in this contribution has yet to be assessed in long-term interactions due to the COVID-19 pandemic. However, in this paper, we have thoroughly detailed our DMS operation and presented the validation of its usability and users’ perception of specific attributes of the robot. As described in Sect. “Decision-making system”, the DMS we propose in this contribution balances satisfying the user’s petitions and the robot’s internal needs. At the same time, the inclusion of user-adaptive mechanisms and an agenda allows the robot to personalize the interaction, improving the system’s performance and the perception of the user. In future work, we would like to test the robot’s behavior in more complex scenarios expanding the robot’s skills and biologically inspired functions.

In future work, we pretend to use the DMS introduced in this contribution to entertainment and therapies to alleviate the cognitive impairment of older adults. For this reason, the usability and user engagement of the system is critical to carrying out these tasks. Furthermore, we plan to conduct more experiments in such a context to test the system’s performance during long-term interactions. These experiments will aim to find the shortcomings of our system and address them by deploying social robots in particular homes that satisfy the user’s needs.

## Data Availability

Data sharing is not applicable to this article since all data is presented in the results section.

## Footnotes

1 Link to video:https://youtube.com/playlist?list=PLXNPQDsfy0lLDQQGuSsI8_hIJhGeVhnDn.

2 Godspeed questionnaire: https://www.bartneck.de/2008/03/11/the-godspeed-questionnaire-series/.
